# Epidemiological evaluation quality of life in patients suffering from early rheumatoid arthritis: a pragmatic, prospective, randomized, blind allocation controlled of a modular program group intervention

**DOI:** 10.4178/epih/e2015048

**Published:** 2015-11-05

**Authors:** Hadi Yousefi, Arvind Chopra, Reza Farrokhseresht, Sanjeev Sarmukaddam, Fariba Asadi Noghabi, Nilima Bedekar, Abdolhosain Madani

**Affiliations:** 1 Faculty of Nursing, Midwifery and Paramedical, Hormozgan University of Medical Sciences, Bandar Abbas, Iran; 2 Center for Rheumatic Diseases, Savitribi Phule Pune University, Pune, India; 3 Department of Internal Medicine, Faculty of Medicine, Hormozgan University of Medical Sciences, Bandar Abbas, Iran; 4 Student Research Committee, Shiraz University of Medical Sciences, Shiraz, Iran; 5 College of Physiotherapy, Sancheti Institute for Orthopedic and Rehabilitation, Pune, India; 6 Social Determinants in Health Promotion Research Center, Hormozgan University of Medical Sciences, Bandar Abbas, Iran

**Keywords:** Epidemiological method, Quality of life, Early rheumatoid arthritis

## Abstract

**OBJECTIVES:**

Epidemiology has taken on new roles in the management of health care services. In this study, we developed a non-pharmacological self-management modular program group intervention and evaluated its efficacy as an adjunct therapy in patients suffering from early rheumatoid arthritis (RA).

**METHODS:**

Patients were randomized to either participate in a non-equivalent intervention group along with the standard of care or only receive standard-of-care treatment at a community rheumatology center. The outcomes measured were a pain visual analog scale (VAS), patient general health (GH) on a VAS, and the Short Form 36 Health Survey version 2 scale measuring quality of life. These parameters were evaluated in the first week to obtain baseline values, and at 20, 32, 48, and 60 weeks to evaluate the efficacy of the intervention group.

**RESULTS:**

The patients were randomized, with 100 patients in the intervention group and 106 in the control group. The intervention and control groups were similar with regard to the percentage of women (86% vs. 89.6%), tobacco usage (25% vs. 19.8%), mean age (42.6±13.2 years vs. 46.6±10.9 years), and disease duration (15.3±6.7 months vs. 14.5±6.6 months). The mean outcomes were significantly different between the two groups, and post-hoc pairwise analysis demonstrated significant deterioration in the control group in contrast to improvement in the intervention group at the second, third, fourth, and fifth evaluations. Improvements were often seen as early as the 12-week and 24-week follow-up visits.

**CONCLUSIONS:**

Epidemiology contributes to the evaluation of how well specific therapies or other health interventions prevent or control health problems. The modular program group intervention implemented in this study appears to be a suitable and feasible method to facilitate much more comprehensive management of early RA in socioeconomically challenged communities.

## INTRODUCTION

Epidemiology has taken on new roles in the management of health care services [1]. When designing and managing health care for a population, it is necessary to manage resources effectively in order to maintain and promote the health of the population. The incidence and prevalence of rheumatoid arthritis (RA) is dynamic and appears to be influenced by both genetic and environmental factors [2]. Many misconceptions exist about RA regarding diet, exercise, and lifestyle, both among the broader community and among RA patients, and patients suffer unnecessarily due to a lack of health information and suitable care [3]. In developing countries, economic limitations are especially important [4,5]. The challenge is to find a cost-effective treatment for better disease control [6]. These findings underscore the complexity of rheumatic diseases and highlight the key role of epidemiological research in understanding these intriguing conditions [7]. Models are useful in guiding epidemiologic research and help determine whether one group is more likely than another to develop a given disease [8]. Epidemiological models of community intervention [9], population-based health management models [10], and multi-state life table models [11] have demonstrated similar consequences in different communities. Non-pharmacological treatment modalities have been used, albeit infrequently, as an adjunct to drug therapy in patients with RA [12]. However, the improvements are variable depending to the type of intervention, and non-pharmacological interventions such as the program described in this study can improve the lives of patients with longer-term illness. The purpose of this pragmatically designed study was to determine whether a self-management modular program group intervention (MPGI) based on interdisciplinary instructions, counseling, and physical therapy in addition to standard-of-care treatment helped achieve better outcomes in patients suffering from early RA. We also assessed whether any such improvements would be maintained over the course of 15 months, with periodic assessments eight, 20, 32, 48, and 60 weeks after the beginning of the intervention. Our study documented improvements in indicators of pain levels (the primary outcome) and in general health and quality of life (the secondary outcomes) in the intervention group when compared with the control group. To our knowledge, this study is the first to evaluate the potential effectiveness of an MPGI in a randomized trial among patients suffering from early RA.

## MATERIALS AND METHODS

### Study design

From January 2011 through January 2013, we conducted a pragmatic, prospective, randomized, assessor-blinded, allocation-controlled study with 15 months of follow-up. It employed a non-equivalent group design in which one group received a combination of active intervention with standard-of-care treatment, while the control group only received the standard of care. Patients attending a community-based rheumatology clinic were screened and, after providing informed consent, were randomized into either the intervention group or the control group. The follow-up visits and assessment were similar for both groups. Prior to the intervention study, a pilot study was conducted on 140 patients to characterize community demographics, disease attributes, clinical data, and the process of patient enrollment; to develop an easy-to-understand and feasible set of booklets and pamphlets for the intervention group; and to assess the suitability, reliability, and internal consistency of the outcome measurement instrument.

### Participants

The inclusion criteria were adults with a disease duration of two years or less who had been diagnosed with RA according to the 1987 criteria of the American College of Rheumatology, were between 18 and 75 years of age, were under supervised outpatient rheumatology care, and were able to read and answer the questionnaires. The exclusion criteria were American Rheumatism Association class IV disease (unable to perform self-care), arthritis other than RA, a positive history of mental illness or alcohol or drug abuse, a medical condition requiring restricted activity (e.g., a history of relatively severe heart, lung, or cerebrovascular disease), previous participation in a similar intervention program in the previous year, and not being fit to participate according to the discretion of the rheumatologist.

**Figure 1.**
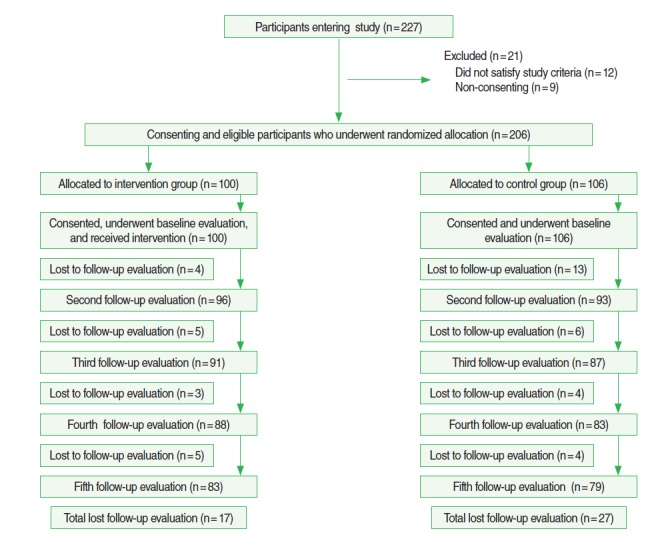
Flow chart of patient recruitment process, randomization and response rates.

A total of 227 patients were considered for the study, and after application of the exclusion criteria, 206 patients were included. Nine patients were excluded due to refusal to participate, four were excluded due to difficulties in reading and writing, and eight were excluded for other reasons. Of the remaining patients, 100 were randomized to the intervention group and 106 were randomized to the control group. Forty-two patients withdrew before completing the entire course of the study, with 83 patients in the intervention group and 79 in the control group completing 15 months of follow-up (final follow-up visit). After the randomized allocation, 13 patients refused to participate further (four in the intervention group and nine in the control group), and 22 patients did not complete the questionnaire (nine in the intervention group and 13 in the control group). Nine patients (four in the intervention group and five in the control group) could not be contacted within the stipulated time (Figure 1).

This study was approved by the ethics committee of the Hormozgan University of Medical Sciences (no. HEC-92-4-25-3).

### Procedures

Early RA patients were informed about this study by personal invitation or on the phone and recruited from a community-based rheumatology clinic (administered through the School of Medicine of Hormozgan University of Medical Sciences) in Bandar Abbas, Hormozgan Province, Iran. This province is located in southern Iran, north of the Persian Gulf, and is divided into 34 towns, 14 islands, 29 rural districts, and 79 villages. Participants were screened by a rheumatologist (Arvind Chopra, Reza Farrokhseresht) and were deemed eligible for this study after providing written informed consent. Subjects were informed of the schedule of visits of the entire study in the first meeting. Subjects were randomized into the intervention and control groups using a four-ball technique, in which a patient was asked (Hadi Yousefi) to remove a ball from a bag of four balls with markings for the intervention group and the control group on two balls each. Subjects were coded (Hadi Yousefi) for the study simultaneously. Subjects filled out the questionnaire under the guidance of the investigators (Hadi Yousefi) during the first visit only. During follow-up visits, before the appointment with the rheumatologist (Reza Farrokhseresht), all patients obtained questionnaires from the reception counter, filled them out independently in a waiting room, and directly submitted them at the reception counter (Fariba Asadi Noghabi). After submission of the questionnaire, they proceeded to a routine rheumatology consultation. The rheumatologist (Reza Farrokhseresht) and the rheumatology nurse (Fariba Asadi Noghabi) at the clinic handled the standard of care for rheumatology outpatients, provided rheumatology services (diagnosis and treatment), and remained blinded to whether patients had been allocated to the treatment or control group throughout the study. Reminders were given two to three weeks in advance of the scheduled visit. In case of absence, a repeat consultation was arranged within a maximum of three days of the scheduled visit. Both groups received reminders on weeks 18, 30, 44, and 56. The intervention group received intervention reminders on weeks 12, 24, 42, and 54. A refresher session consisted of four weekly workshops was conducted in the 32nd week in the intervention group. Each group received one session weekly for 2.5 hours during the four weeks which covering the all material of the MPGI.

### Standard-of-care medical treatment

Standard-of-care medical treatment was provided by experienced rheumatology team members. No additional training was needed. A program manual was developed from observation of the existing program. All patients were evaluated by a rheumatologist as part of their medical treatment, while rheumatology nurses and paramedics handled patient-centric issues of function and quality of life measurement and advice. They also helped provide logistical support.

### Modular program group intervention

The MPGI was based on interdisciplinary instruction, counseling, and physical therapy (exercises). The patients in the intervention group were divided in 10 groups of 10 patients each. Each group received one session weekly for 2.5 hours during the first eight weeks of the study. The intervention meetings consisted of eight weekly workshops for each group spread over eight weeks. During the first two weeks, the sessions focused on improving the knowledge, attitudes, and practices of patients with RA. The third workshop was on pain management. The groups received two sessions covering exercise, physical therapy programs, and joint protection in the fourth and fifth week. In the sixth week, the session dealt with nutrition and healthy diets. A session dealing with fatigue control and stress management was conducted in the seventh week. A review session covering the material of all of the above workshops was conducted in the eighth week. The meetings were highly interactive, focusing on building skills, sharing experiences, and providing support in addition to the scheduled intervention programs. The subjects were provided a package about arthritis research and arthritis care (booklets and 10 pamphlets).

### Evaluation

A baseline evaluation was carried out in the first week, followed by evaluations at 20, 32, 48, and 60 weeks.

### Sample size

The sample size was calculated for the pain visual analog scale (VAS), as it was the most important parameter evaluated in the study. Reduction in the pain VAS was considered to be the primary variable of interest. The sample size corresponding to 7% reduction in the pain VAS [13] with a power of 80% and a significance level of 5%.

### Analysis

Standard rheumatology forms were used to obtain clinical data. Patient-centric data, including the outcomes of all questionnaires, drew on patient-reported outcomes and supervised interviews. The patient-reported outcomes included general health over the past week reported on a VAS and pain at rest over the past week reported on a VAS, and were recorded by the patient and physician. Version 2 of the Short Form Health Survey (SF-36) was used with permission [14]. It contains a physical component scale (PCS) and a mental health component scale (MCS), which jointly measure the following health domain scales: physical functioning (PF); physical role (PR); bodily pain (BP) severity; general health (GH); vitality (VT); social function (SF); emotional role (ER); mental health (MH).

The laboratory tests included the erythrocyte sedimentation rate and C-reactive protein levels. Rheumatoid factor was considered to be positive if the reference values used by the local laboratory indicated positive values at any time over the course of the disease. We also collected information on arthritis medication usage.

Data processing and analysis were conducted using system 2.0 of the Biomedical Data Package, version 7.0 (BMDP Co., Berkeley, CA, USA). The mean scores of the clinical assessments, health status, and quality of life scales used in the analyses were expressed as the percentage of the predicted values based on the subject-specific sex, residency, smoking tobacco, pain killer and family size. The normality of the variables was tested by the Shapiro-Wilk test. The following additional statistical tests were performed as appropriate: general linear model-repeated measures analysis of variance, post-hoc testing, pairwise comparisons with the Bonferroni adjustment, and estimated marginal mean values of the scales at each evaluation stage pertaining to a given objective. These analyses were repeated with age, disease duration, sex, and years of education as covariates. Subsequently, the cross-tabulation of each explanatory variable on the outcome variable was carried out and tested using the chi-square test for categorical variables and the Student’s t-test and Man-Whitney U-test for quantitative data. Correlation coefficients were also estimated for appropriate pairs.

## RESULTS

No significant differences existed between the groups at baseline (Table 1). Patients in both arms of the interventional study exhibited significant improvement (often p<0.05) at the 60-week endpoint for several clinical variables, the pain VAS, the GH VAS, and the SF-36 physical and mental components and the eight domains thereof. Improvements were often seen as early as the 12-week and 24-week follow-up visits (Table 2).

Of particular note is the fact that no significant differences at baseline were found between the study groups regarding the use of pain medication (Table 1). It can be concluded that the prevalence of pain medication use in the intervention and control groups was similar, with a few individual exceptions due to chance results of the randomized allocation process.

A post-hoc pairwise analysis using the Bonferroni adjustment between the evaluation visits was done, showing significant deterioration in the control group in contrast to improvement in the intervention group in the mean values of the pain VAS, GH VAS, PCS, MCS and most of the eight domains of the SF-36 (Table 3).

Significant differences were found between the intervention and control groups with regard to the GH VAS, the PCS and MCS, and the PF, PR, VT, ER scores at the third, fourth, and fifth evaluations; GH and MH at the fourth and fifth evaluations; and SF at the second, fourth, and fifth evaluations, with higher mean values observed in the intervention group. Significant between-group differences were observed in the pain VAS and the BP domain at the second, third, fourth, and fifth evaluations, with lower mean values observed in the intervention group (Tables 2 and 4). Therefore, the self-management program had an observable impact on the PCS, MCS, and the eight domains of the SF-36 in the intervention group.

## DISCUSSION

The present study demonstrated that the intervention group displayed clinically impressive and often statistically significant (p<0.05) improvements in RA outcomes, both short-term and long-term, for physician and patient global health assessments, the PCS and MCS components of the SF-36, and the eight domains of the SF-36 scores, whereas the control group did not.

Considerable advances have recently been made in the pharmacological treatment of RA, but it is associated with increased toxicity and cost and the long-term outcomes still remain unknown. Non-pharmacological treatment is cost-effective, has less toxicity, and has been associated with better long-term outcomes [15].

Despite substantial advances in the medical management of RA, it continues to be difficult to treat and has a considerable effect on the lives of patients [16]. The majority of patients (75%) do not achieve full remission and 15% have a sustained high or moderate level disease activity in the first three years after the onset of the disease [17]. The burden of RA and its association with impaired quality of life have become an international health priority addressed by initiatives such as the Bone and Joint Decade launched by the World Health Organization [18].

Several non-pharmacological clinical experimental methods and/or models [19-21] have been evaluated in patients suffering from arthritis in general, and RA in particular, to address the question of how methods in addition to standard-of-care therapy can be used to address the needs of patients [22].

This community clinical study was focused on the role of a planned, structured, non-pharmacological MPGI in patients suffering from early RA who were treated with standard-of-care treatment in a community rheumatology clinic. Considerable evidence exists to support the recommendation of multimodular non-pharmacological interventions, such as self-management programs, for community or clinical settings [20].

Unimodular interventions such as exercise [23], cognitive therapies [24], and orthoses [12] show promise but need further research. The potential role of dietary modifications in treating RA is exciting [25], but needs further study before guidelines can be created. These interventions can serve as a cost-effective strategy complementing standard treatment. The challenge henceforth is to further investigate these interventions and how they may be tailored to meet different cultural and individual needs. The timing and duration of these options are likewise still a matter for research.

A 2009 review by Vliet Vlieland & Pattison [12] summarized the available evidence on the effectiveness of non-pharmacological therapies for early RA. The effectiveness of multidisciplinary team-care programs, specialist nursing care, electro-physical modalities (including passive hydrotherapy), wrist orthoses, and dietary interventions has not been adequately studied in patients with early RA. The results of the current exploratory study provide novel information about non-pharmacological interventions and introduce a new model MPGI for patients suffering from early RA.

The American College of Rheumatology has published explicit management guidelines [26,27] and described a “window of opportunity” in the very early period of clinical RA that can be effectively targeted to control the disease, preventing articular deformities and other eventual complications of RA. Several studies thereafter have demonstrated excellent therapeutic responses in patients with early RA [26,28].

The present study found significant differences between the intervention and control groups with regard to the mean pain VAS scores at the second, third, fourth, and fifth evaluations (with lower values in the intervention group) and the general health VAS scores at the third, fourth, and fifth evaluations (with higher values in the intervention group). This indicates that the MPGI had an observable impact on the pain VAS and general health VAS in the intervention group. A pairwise analysis showed significant reductions in the pain VAS and significant improvements in the general health VAS that continued for an extended period of time (after the first and second evaluations) in the intervention group. The reduction of the 25th and 75th percentile pain VAS values and the general health VAS in the intervention group to values of 20 and less in the intervention group at the time of study completion was impressive and further supports the positive impact of the MPGI evaluated in this study.

Several studies using multimodular programs have made similar observations in diverse groups of subjects, noting a significant decrease in the pain VAS and improvements in general health [19,21,24].

The results of the current study demonstrated significant differences between the intervention and control groups with regard to the SF-36 PCS, MCS, and its eight domains at the third, fourth, and fifth evaluations, with higher values found in the intervention group than in the control group. Measures such as the SF-36 provide the building blocks for creating models of quality of life, and have been used to evaluate different perspectives on this parameter across patients with a range of chronic physical conditions as well as healthy individuals. Within this framework, the use of generic measures of quality of life can offer opportunities to frame research and interventions that appropriately target the quality of life of individuals with RA [18].

In a randomized control trial, Goeppinger et al. [29] showed that improvements were achieved in functional disability measures as well as self-related health and social role limitations. Kennedy et al. [30] showed significant improvement in social role limitations and functional disability in their longitudinal observational study of a self-management program. Our findings confirm this pattern and demonstrate the wide range of parameters influenced by the multimodular program that we evaluated.

Our study had several limitations. Only patients whose disease had lasted for less than two years were included, and our results may therefore have limited applicability to patients with chronic RA and rheumatoid deformities. The patients were requested not to discuss their treatment allocation with the rheumatology team, but it is possible that some patients from the intervention and control groups exchanged information regarding the MPGI. The therapeutic approach advocated in the current research study may still require important modifications and re-evaluation.

In conclusion, these findings have implications for health policy and the allocation of funding for both health care and research. Our results can be used to construct preventive instructional non-pharmaceutical strategies to treat RA in ways suited to specific communities.

## Figures and Tables

**Table 1.** Baseline evaluation of patients suffering from early rheumatoid arthritis

	Intervention MPGI (n = 100)	Control (n = 106)	p-value
Weight (kg)	61.09 ± 10.31 (61.00)	61.64±7.49 (61.50)	0.66^1^
Disease duration (mo)	15.29±6.73 (17.50)	14.52±6.67 (14.50)	0.49^1^
Duration of schooling (yr)	7.60±5.18 (5.00)	6.51 ±5.28 (5.00)	0.10^1^
Age (yr)	42.90± 13.24 (40.00)	46.60± 10.97 (48.00)	0.07^1^
Family size (n)	4.21 ±1.42 (4.00)	5.01 ±2.29 (5.00)	0.05^1^
Sex			0.28^2^
Female	86 (86.0)	95 (89.6)	
Male	14 (14.0)	11 (10.4)	
Residency			0.35^2^
Urban	75 (75.0)	83 (78.3)	
Rural	25 (25.0)	23 (21.7)	
Smoking			0.23^2^
Yes	25 (25.0)	21 (19.8)	
No	75 (75.0)	85 (80.2)	
RF (normal value= <40 U/mL)	137.63 ±119.37	122.79± 103.20	0.34^1^
CRP (normal value = 0.0-0.8 mg/dL)	48.04± 28.78	45.65± 24.34	0.90^1^
MTX dosage (mg/wk)	10.5± 4.71	10.04 ±5.11	0.51
Steroid dosage (mg/d)	5.32±1.10	5.21 ±1.20	0.47
Pain killer			0.44
Yes	18 (18.0)	21 (19.8)	
No	82 (82.0)	85 (80.2)	

Values are presented as mean±standard deviaton (median) or number (%). MPGI, modular program group intervention; RF, rheumatoid factor; CRP, C-reactive protein; MTX, methotrext.

1 p-value by Student’s t-test or Mann-Whitney U-test.

2 p-value by chi-square test.

**Table 2.** Comparing mean values of the clinical and quality of life (SF-36 component) variables at each evaluation stage

	1 st (baseline)		p-value^1^	2nd (5 mo)		p-value^1^	3rd (8 mo)		p-value^2^	4th (12 mo)		p-value^2^	5th (15 mo)		p-value^2^
	Intervention (n = 100)	Control (n = 106)		Intervention (n = 96)	Control (n = 93)		Intervention (n = 91)	Control (n = 87)		Intervention (n = 88)	Control (n = 83)		Intervention (n = 83)	Control (n = 79)	
GH (VAS)^3^	43.7(11.4)	43.7(13.1)	0. 99	29.9(10.9)	31.7(10.0)	0.23	21.6(8.5)	33.72(11.4)	<0.001	21.8 (8.6)	31.0(10.1)	<0.001	16.1 (6.8)	30.9(10.9)	<0.001
Pain (VAS)^4^	48.5(14.0)	52.0(12.2)	0. 06	27.8 (9.7)	24.1 (8.7)	0.007	19.5(7.7)	34.1 (9.7)	<0.001	21.4(8.1)	25.8(10.0)	0.002	16.1 (6.4)	29.1 (10.0)	<0.001
PCS^5^	61.72(9.42)	60.26 (9.46)	0.27	61.95(11.62)	60.94 (8.54)	0.50	62.78 (7.90)	55.64 (9.34)	<0.001	67.09 (8.02)	52.98(10.94)	<0.001	70.63 (8.56)	49.80(11.58)	<0.001
MCS^5^	59.46 (9.84)	60.84(10.25)	0.32	60.48(12.80)	61.38 (9.96)	0.59	63.49(10.33)	55.16(11.06)	<0.001	63.13(9.92)	52.30 (7.79)	<0.001	68.45 (11.08)	46.63 (8.43)	<0.001

SF-36, Short Form 36 Health Survey; Values are presented as mean (standard deviation). GH, general health; VAS, visual analog scale; PCS, physical component scale; MCS, mental component scale.

1 Man Whitney U-test.

2 Student’s t- test.

3 Score 0-100 at 0 for perfect health (no disease activity) and 100 for intensely weak health (highest disease activity possible).

4 Score 0-100 mm at the ‘0’ mark, it says ‘no pain at all’, and at the ‘100’ mark, ‘pain as bad as it could be.’

5 Range is 0-100 (worst=0, best=100) with higher scores indicating higher levels of function and or better health.

**Table 3.** Repeated measures analysis of variance for pair wise comparisons in mean values of the clinical and quality of life variables (SF-36 component) at each evaluation stage

Pairwise compaarison	GH (VAS)^1^		Pain (VAS)^2^		PCS^3^		MCS^3^	
	Intervention	Control	Intervention	Control	Intervention	Control	Intervention	Control
1st evaluation and 2nd evaluation	13.5 (< 0.001)	11.1 (< 0.001)	21.2 (< 0.001)	28.9 (< 0.001)	-0.3(1.00)	-0.8 (1.00)	-1.738(1.00)	-1.840(1.00)
1st evaluation and 3rd evaluation	22.5 (< 0.001)	9.2 (<0.001)	30.2 (< 0.001)	17.3 (< 0.001)	-1.1 (1.00)	4.4 (0.04)	-6.048 (< 0.001)	3.935 (0.18)
1st evaluation and 4th evaluation	21.7 (< 0.001)	12.4 (< 0.001)	28.6 (< 0.001)	25.8 (< 0.001)	-5.6 (< 0.001)	6.5 (<0.001)	-5.163 (0.006)	6.431 (< 0.001)
1st evaluation and 5th evaluation	27.2 (< 0.001)	11.9 (< 0.001)	34.0 (< 0.001)	22.7 (<0.001)	-8.8 (< 0.001)	10.1 (< 0.001)	-10.839 (< 0.001)	12.385 (< 0.001)
2nd evaluation and 3rd evaluation	9.0 (< 0.001)	-1.9(1.00)	9.1 (< 0.001)	-11.5 (<0.001)	-0.7(1.00)	5.2 (0.003)	-4.310(0.13)	5.776 (<0.001)
2nd evaluation and 4th evaluation	8.2 (< 0.001)	1.3(1.00)	7.4 (< 0.001)	-3.0 (0.20)	-5.3 (0.005)	7.3 (< 0.001)	-3.425 (0.68)	8.272 (<0.001)
2nd evaluation and 5th evaluation	13.7 (< 0.001)	0.8 (1.00)	12.8 (< 0.001)	-6.2 (<0.001)	-8.5 (< 0.001)	10.9 (< 0.001)	-9.101 (<0.001)	14.225 (< 0.001)
3rd evaluation and 4th evaluation	-0.8(1.000)	3.2 (0.28)	-1.7(1.00)	8.5 (<0.001)	-4.6 (< 0.001)	2.1 (1.00)	0.885(1.00)	2.496 (1.00)
3rd evaluation and 5th evaluation	4.7 (< 0.001)	2.7 (0.54)	3.7 (< 0.001)	5.3 (< 0.001)	-7.8 (< 0.001)	5.7(0.012)	-1.738(1.00)	-1.840(1.00)
4th evaluation and 5th evaluation	5.5 (< 0.001)	-0.5(1.00)	5.4 (< 0.001)	-3.2 (0.47)	-3.2 (0.007)	3.6(0.21)	-6.048 (< 0.001)	3.935 (0.18)

Values are presented as mean difference (p-value). The p-values calculated by adjustment for multiple comparisons (Bonferroni). GH, general health; VAS, visual analog scale; PCS, physical component scale; MCS, mental component scale.

1 SF-36, Short Form 36 Health Survey; Score 0-100 at 0 for perfect health (no disease activity) and 100 for intensely weak health (highest disease activity possible).

2 Score 0-100 mm at the ‘0’ mark, it says ‘no pain at all’, and at the ‘100’ mark, ‘pain as bad as it could be.’

3 Range is 0-100 (worst=0, best=100) with higher scores indicating higher levels of function and or better health.

**Table 4.** Comparing mean values of the quality of life (SF36-8 domains) variables at each evaluation stage

Variable^1^	1st (baseline)		p-value	2nd (5 mo)		p-value	3rd (8 mo)		p-value	4th (12 mo)		p-value	5th (15 mo)		p-value
	Intervention (n = 100)	Control (n = 106)		Intervention (n = 96)	Control (n = 93)		Intervention (n = 91)	Control (n = 87)		Intervention (n = 88)	Control (n = 83)		Intervention (n = 83)	Control (n = 79)	
PF	62.03(11.39)	62.38 (20.49)	0.88	66.05 (14.43)	65.60(10.66)	0.81	64.58(15.19)	60.12(8.76)	0.02	70.01 (12.23)	58.07 (6.28)	<0.001	75.05(10.64)	53.58 (8.45)	<0.001
PR	67.37 (17.77)	67.74(10.60)	0.85	71.09(15.25)	68.96(11.96)	0.31	67.50(11.93)	62.56 (9.99)	0.003	70.81 (8.68)	64.98(10.02)	<0.001	75.03 (8.93)	60.58 (9.77)	<0.001
BP	44.42 (12.81)	14.93(0.86)	0.86	30.64(7.31)	24.90 (9.90)	<0.001 22.46(10.85)		31.15(10.11)	<0.001	23.69 (8.22)	27.69(8.15)	0.002	18.44(6.99)	29.22 (9.24)	<0.001
GH	56.92 (11.47)	57.69(18.44)	0.72	60.25 (14.37)	60.81 (11.92)	0.77	59.06(13.65)	56.59(12.45)	0.21	64.04 (10.84)	52.91 (9.76)	<0.001	70.51 (10.75)	50.07 (7.72)	<0.001
VT	58.41 (13.90)	60.87 (11.82)	0.17	55.95 (12.82)	58.29(11.99)	0.20	58.66(13.50)	53.77(11.50)	0.01	62.97 (14.61)	55.75(10.58)	<0.001	69.08(10.56)	50.31 (8.10)	<0.001
SF	68.45 (15.74)	65.77 (21.10)	0.30	66.56(14.62)	62.02 (9.79)	0.01	67.22(18.25)	64.83(13.94)	0.33	69.63 (15.84)	62.24(17.97)	0.005	76.26(17.05)	54.05 (7.93)	<0.001
ER	78.96(18.68)	75.61 (12.74)	0.08	75.00(18.68)	71.45 (16.47)	0.17	81.15(17.52)	73.39(10.20)	<0.001	82.03 (11.89)	71.35(10.43)	<0.001	84.49(11.58)	66.26(11.49)	<0.001
MH	64.45 (13.26)	66.56(18.99)	0.36	60.74(12.10)	61.75 (10.48)	0.54	61.42(12.10)	62.57(13.20)	0.46	65.14(18.85)	55.87(11.68)	<0.001	68.23(14.99)	49.84(10.15)	<0.001

Values are presented as mean (standard deviation). The p-value by Man Whitney U- test. SF-36, Short Form 36 Health Survey; PF, physical functioning; PR, physical role; BP, bodily pain severity; GH, general health; VT, vitality; SF, social function; ER, emotional role; MH, mental health.

1 Score is 0-100 where 0 is denoting poor and 100 is denoting high.
